# Mental health law and the UN Convention on the rights of persons with disabilities

**DOI:** 10.1016/j.ijlp.2013.11.024

**Published:** 2014-05

**Authors:** George Szmukler, Rowena Daw, Felicity Callard

**Affiliations:** a Institute of Psychiatry, King's College London De Crespigny Park, London, UK; b Legal Consultant; formerly Royal College of Psychiatrists, London, UK; c Durham University, Durham, UK

**Keywords:** Rights, Disabilities, Mental illness, Involuntary treatment, CRPD

## Abstract

People with a mental illness may be subject to the UN Convention on the Rights of Persons with Disabilities (CRPD), depending on definitions of terms such as ‘impairment’, ‘long-term’ and the capaciousness of the word ‘includes’ in the Convention's characterisation of persons with disabilities. Particularly challenging under the CRPD is the scope, if any, for involuntary treatment.

Conventional mental health legislation, such as the Mental Health Act (England and Wales) appears to violate, for example, Article 4 (‘no discrimination of any kind on the basis of disability’), Article 12 (persons shall ‘enjoy legal capacity on an equal basis with others in all aspects of life’) and Article 14 (‘the existence of a disability shall in no case justify a deprivation of liberty’).

We argue that a form of mental health law, such as the Fusion Law proposal, is consistent with the principles of the CRPD. Such law is aimed at eliminating discrimination against persons with a mental illness. It covers all persons regardless of whether they have a ‘mental’ or a ‘physical’ illness, and only allows involuntary treatment when a person's decision-making capability (DMC) for a specific treatment decision is impaired — whatever the health setting or cause of the impairment — and where supported decision making has failed. In addition to impaired DMC, involuntary treatment would require an assessment that such treatment gives the person's values and perspective paramount importance.

## 1 Introduction

This paper addresses the question of whether involuntary treatment of persons with a mental illness is compatible with the United Nations Convention on the Rights of Persons with Disabilities (CRPD), and if so, in what circumstances.

Relevant aspects of the CRPD will be described, focussing on the challenges it presents to involuntary treatment. It is proposed that the principles underlying a form of law, known as the ‘Fusion Law’, are consistent with the CRPD. The Fusion proposal was animated by the aim of countering the discrimination that is inherent in conventional forms of mental health legislation. It allows for involuntary treatment under certain, tightly circumscribed conditions. These involve considerations of ‘decision-making capability’ and a version of ‘best interests’, concepts subject to different understandings, but that we will define so far as necessary to progress the argument.

## 2 The UN Convention on the rights of persons with disabilities

The CRPD was passed by the General Assembly of the United Nations in December 2006. By February 2013, it has been signed by 155 countries and ratified by 127. It sets out key rights that citizens with a disability should enjoy in a fair society. It is one of the nine core human rights treaties of the UN. The overall purpose, stated in Article 1, is to “promote, protect and ensure the full and equal enjoyment of all human rights and fundamental freedoms by all persons with disabilities, and to promote respect for their inherent dignity”. The elimination of discrimination by ensuring that rights may be enjoyed on an equal basis with others is a fundamental aim. While, arguably, most of the rights in the CRPD are already protected by other UN treaties, the CRPD frames rights in a way that is specific for people with disabilities, a group of persons rarely referred to in those other treaties (Bartlett, 2012, Lawson, 2006). Noteworthy was the formal, active involvement of disabled people's organisations, including the World Network of Users and Survivors of Psychiatry, in the drafting and negotiations behind the CRPD.

‘Disability’ is not formally defined in the CRPD, allowing individual State Parties considerable latitude in how they define disability in their domestic law. People with disabilities are characterised as follows: *Persons with disabilities include those who have long-term physical, mental, intellectual or sensory impairments which in interaction with various barriers may hinder their full and effective participation in society on an equal basis with others*. The use of the word ‘include’ in the statement above allows for a non-exhaustive description of ‘disability’ that is not settled; neither are the meanings of terms such as ‘long-term’ and ‘impairments’. It is accepted by the Committee on the Rights of Persons with Disabilities that people with a ‘mental illness’ (referred to as having a ‘psychosocial disability’) fall under the Convention. Whether all people with a ‘mental illness’ are appropriately considered as having a ‘disability’ is a moot question. It raises larger philosophical and sociological issues as regards what it means to draw together the domains of mental health and disability (and mental health politics and disability politics). For instance: does someone who has intermittent and short-lived episodes of mental distress and disability, constituting temporary departures from their ‘normal self’ (when they have no diagnosable mental illness nor disability), have a ‘disability’ in line with the CRPD? What is the nature of the ‘impairment’ in mental illness? If a person receives any diagnosis from (any) classificatory manual of mental disorder, are they *de facto* a person with a disability under the CRPD? Can the discrimination consequent on being diagnosed with a mental illness constitute a disability? Do people with mental illness identify with the idea that they are ‘persons with disabilities’? Is the ‘social model of disability’ — which as we shall see later, clearly shapes the definition of persons with disabilities in the CRPD — adequate as regards the conceptualization of mental illness and mental distress? Discrimination against people with mental illness is certainly rife; but to what extent is it best explored through the prism of disability in contrast to, for example, stereotypes involving ‘dangerousness’? These are important unsettled, questions, but for the purposes of this paper we set them aside and accept that, at the very least, people with a long-term serious mental illness will fall within the scope of the CRPD.

The CRPD contains the classic array of civil and political rights, such as the right to liberty (Article 14) and integrity of the person (Article 17), rights to freedom of expression (Article 21) and privacy (Article 22), the right to freedom from torture and inhuman treatment (Article 15), and rights to equal recognition before the law (Article 12) and access to justice (Article 13). It also includes economic, social and cultural rights that have come to prominence since the Second World War, including the right to home and family life (Article 23), the right to education (Article 24), and rights to health (Article 25), habilitation and rehabilitation (Article 26). Some of these rights have been framed so as to have particular relevance to people with disabilities: the right to non-discrimination (Article 5), the right to independent living and community inclusion (Article 19), the right to personal mobility (Article 20), the right to work and employment (Article 27), the right to participation in cultural life (Article 30) and the right to be free from exploitation and abuse (Article 16).

Countries are placed under a variety of obligations to take measures to modify or abolish existing discriminatory laws, regulations and practices, as well as to provide programmes to support the rights of persons with disabilities (Article 4). These obligations include, for example, a duty to provide appropriate training regarding disability issues to those involved in the administration of justice (Article 13), concrete programmes to assist people with disabilities and their caregivers to recognise and combat exploitation (Article 16), obligations to provide community support services (Article 19), and overarching duties on states to raise awareness of disability issues (Article 8) and to combat discrimination (Article 5).

Appendix A sets out in full those Articles (12, 14, 17, 25) that will be most frequently referred to in our discussion of whether involuntary treatment can be consistent with the CRPD.

The Convention establishes the UN Committee on the Rights of Persons with Disabilities. States Parties are required on a periodic basis to report to the Committee on their progress in implementing the Convention, and the Committee in turn publishes comments about this progress. Crucially, Article 33 of the Convention requires governments to ensure that representatives of civil society, in particular persons with disabilities and their representative organisations, are fully involved in monitoring the implementation of the Convention. Furthermore, for states that have ratified the optional protocol, individuals who consider themselves victims of violations of the Convention will be able to make formal complaints for determination by the Committee.

In some countries that have ratified the CRPD, such as the UK and other case law countries, the Convention is not part of its domestic law unless incorporated into law by legislation. So it is not binding on its domestic courts, but like any other international convention to which a state is party, it can be referred to by courts and can be used to interpret domestic law. In many other ratifying countries the Convention is binding as part of domestic law. The reports of the Committee on national progress in implementation will be public, and may create political pressures nationally and internationally. When a State Party has signed the Optional Protocol, a side agreement to the Convention, it recognises the competence of the Committee on the Rights of Persons with Disabilities to examine complaints from individuals. As of February 2013, there were 90 signatories and 76 parties to this Optional Protocol. For individual complaints, determinations also will be public. The United States has signed, but not ratified, the CRPD; China has both signed and ratified. How effective the Committee on the Rights of Persons with Disabilities will prove remains to be seen.

## 3 Involuntary treatment and the CRPD

One aspect of the CRPD appears to be particularly challenging to conventional mental health practice. This concerns involuntary treatment. Along with the general right to liberty, similar to that contained in other human rights instruments, it provides that *‘the existence of a disability shall in no case justify a deprivation of liberty.’* (Art. 14(1)(b)). The Office of the UN High Commissioner for Human Rights adopts a robust view of this provision, as it applies to psychiatric detention:

*‘[48.] … Article 14, paragraph 1 (b), of the Convention unambiguously states that “the existence of a disability shall in no case justify a deprivation of liberty”. Proposals made during the drafting of the Convention to limit the prohibition of detention to cases “solely” determined by disability were rejected. As a result, unlawful detention encompasses situations where the deprivation of liberty is grounded in the combination between a mental or intellectual disability and other elements such as dangerousness, or care and treatment. Since such measures are partly justified by the person’s disability, they are to be considered discriminatory and in violation of the prohibition of deprivation of liberty on the grounds of disability, and the right to liberty on an equal basis with others prescribed by article 14’.*^1^

On this account, ‘mental disorder’ or ‘mental illness’, even if it comprises only one of a number of necessary criteria for involuntary detention, makes that set of criteria incompatible with Article 14, that a disability shall in no case justify a deprivation of liberty.

The Committee on the Rights of Persons with Disabilities in 2011 in its ‘concluding observations’ following consideration of reports submitted by Spain^2^ and Tunisia^3^ echoed this interpretation.

For Spain:

*’36. The Committee recommends that the State Party: review its laws that allow for the deprivation of liberty on the basis of disability, including mental, psychosocial or intellectual disabilities; repeal provisions which authorise involuntary internment linked to an apparent or diagnosed disability; and adopt measures to ensure that health care services including all mental health care services are based on informed consent of the person concerned’*;

and, for Tunisia:

*25. The Committee recommends that the State party repeal legislative provisions which allow for the deprivation of liberty on the basis of disability, including a psychosocial or intellectual disability.*

*28. The Committee is concerned about the lack of clarity concerning the scope of legislation to protect persons with disabilities from being subjected to treatment without their free and informed consent, including forced treatment in mental health services.*

We shall return to the comments concerning informed consent later, but it is clear that these interpretations place current involuntary treatment regimes under increasing scrutiny.

Minkowitz, 2006, Minkowitz, 2011 argues that involuntary treatment is ruled out entirely. She argues Article 12, that persons shall *‘enjoy legal capacity on an equal basis with others in all aspects of life’*, by making no explicit reference to ‘substitute decision making’ in any of its subsections, rejects it. Earlier drafts of Article 12 did make reference to substitute decision making, but these were dropped because agreement could not be reached about the implications of its inclusion. Minkowitz (2011) states: “*A provision recognizing substituted decision‐making would have overcome the general principle of equal legal capacity, and the obligation to ensure that measures related to legal capacity respect the will and preferences of the person, constituting an explicit exception. In the absence of such an exception, the plain meaning must prevail without reading in the exception that was rejected”*. She also interprets Article 14 as did the Commissioner for Human Rights in the quotation given above. Article 17, in recognising that persons with disabilities have the *‘right to respect for physical and mental integrity on an equal basis with others*’, Minkowitz argues, prevents treatment being given without consent. She points out that the CRPD Reporting Guidelines for Article 17 require State Parties to report on measures taken to protect persons with disabilities from medical (or other) treatment given without free and informed consent. The ‘Concluding Observations’ from the Committee on Spain and Tunisia appear to support her point. Arguing further, Minkowitz (2011) points out that Article 25(d) requires that health professionals *‘provide care of the same quality to persons with disabilities as to others, including on the basis of free and informed consent….* ‘, and that reading Article 25 in conjunction with Article 12 indicates that the “consent of third parties is not substituted for that of persons with disabilities, who at all times enjoy the right to exercise legal capacity according to their own will and preferences”.

Some background may help an understanding of the key issues here (for details, see Dhanda, 2006). One of the most important aims of the CRPD is the elimination of discrimination against people with disabilities. Especially requiring remedy is the loss for many disabled persons of their rights, often encompassing virtually every sphere of their lives, to act according to their choices and preferences following their placement under forms of guardianship. Decisions are then made for them according to judgements and preferences that are not theirs. It is clear that in very many instances the legal criteria and safeguards governing such guardianship are highly unsatisfactory, for example, orders being made without the knowledge of the person subject to them, removing automatically a large array of rights, having no defined time limit or provisions for appeal (Drew et al., 2011). The social model of disability adopted by the CRPD holds that ‘disability’ is not an attribute located within an individual but that it ‘results from the interaction between persons with impairments and attitudinal and environmental barriers that hinders their full and effective participation in society on an equal basis with others’, and that with support of varying degrees, these significant barriers can be counteracted. Thus State Parties are required to provide a raft of actions to remove the barriers, including the abolition of discriminatory laws.

Undoubtedly, in relation to long-term disabilities, such as those resulting from sensory or intellectual impairments or chronic health conditions, the proposed measures to counter discrimination are highly appropriate. However, what is not clear is how far this thinking applies to persons who experience sudden or potentially short-term impairments — often reversible with treatment, social support or elapsing of time — that significantly affect their decision-making capabilities. In this paper we are especially concerned about treatment decisions carrying the potential for extremely serious consequences. There are two issues here. The first concerns whether ‘disability’ as characterized in the CRPD applies to shorter-term impairments of decision-making capability. Is there a distinction to be made between this kind of impairment and those more conventionally associated with ‘disabilities’? If there is a distinction, how does it apply to those who do have a long-term disability, for example, an intellectual disability, but who also have an impairment of treatment decision-making capability, either short-term (that is, superimposed on a long-term disability) or long-term? The second issue is under what circumstances, if any, it would be appropriate for another person, duly appointed, to make decisions on behalf of a person with a significantly impaired decision-making capability and how such a judgement should be made.

The second issue caused much controversy in the development of the CRPD, especially the framing of what eventually became Article 12, dealing with ‘legal capacity’ — that persons shall ‘enjoy legal capacity on an equal basis with others in all aspects of life’. Direct reference to the possibility of decision-making by another person on behalf of a disabled person, even as a last resort, is not made, though there were arguments that it should be. Article 12(4), the result of attempts at achieving consensus, perhaps by remaining silent on some issues, is ambiguous but can, arguably, be read as implying that such interventions are not entirely excluded. It refers to safeguards that

*‘......shall ensure that measures relating to the exercise of legal capacity respect the rights, will and preferences of the person, are free of conflict of interest and undue influence, are proportional and tailored to the person*'*s circumstances, apply for the shortest time possible and are subject to regular review by a competent, independent and impartial authority or judicial body. The safeguards shall be proportional to the degree to which such measures affect the person*'*s rights and interests’.*

These ‘safeguards’ might suggest the possibility of something beyond safeguarding supported decision-making.

We shall argue that there is an important conceptual distinction to be made between DMC for a time and task specific treatment decision as opposed to a ‘disability’. Furthermore, that we need to recognise that there are circumstances of significantly impaired DMC where despite best efforts at supported decision-making, this is not possible. In such circumstances, treatment that is administered without the consent of the person might on occasion be justifiable. We propose that a form of ‘capability-based’ law, narrowly drawn, is consistent with the CPRD in providing for involuntary treatment that is non-discriminatory.

## 4 A clinical–empirical argument that a comprehensive impaired DMC-based law is consistent with the principles underlying the CRPD

The following conditions would need to be met for a ‘disability-neutral’ mental health law under the terms of the CRPD

• ‘Respect for inherent dignity, individual autonomy, including the freedom to make one's own choices, and independence of persons’. (Article 3 (a))
• No ‘discrimination of any kind on the basis of disability’ (Article 4).
• Persons shall ‘enjoy legal capacity on an equal basis with others in all aspects of life’ (Article 12.2).
• The ‘existence of a disability shall in no case justify a deprivation of liberty’ (Article 14.1 (b))
• ‘Every person with disabilities has a right to respect for his or her physical and mental integrity on an equal basis with others’ (Article 17)
• Health professionals to provide ‘care of the same quality to persons with disabilities as to others, including on the basis of free and informed consent’ (Article 25(d))

Clearly mental health legislation as exemplified by the MHA 1983 (amended in 2007) does not meet the requirements of the CRPD. First, involuntary treatment is based on ‘status’, that is, suffering from a ‘mental disorder’ — that is, a form of disability under the terms of the CRPD. Second, this status is then coupled with a ‘risk’ criterion — posing a danger to self or to others. Third, except in a few instances, involuntary treatment can be given without taking into account the patient's right to exercise legal capacity. It is presumably precisely this form of legislation that the Office of the UN High Commissioner for Human Rights was referring to when concluding that States Parties

*[49] “must ……. repeal … provisions authorizing institutionalization of persons with disabilities for their care and treatment without their free and informed consent, as well as provisions authorizing the preventive detention of persons with disabilities on grounds such as the likelihood of them posing a danger to themselves or others, in all cases in which such grounds of care, treatment and public security are linked in legislation to an apparent or diagnosed mental illness. This should not be interpreted to say that persons with disabilities cannot be lawfully subject to detention for care and treatment or to preventive detention, but that the legal grounds upon which restriction of liberty is determined must be de-linked from the disability and neutrally defined so as to apply to all persons on an equal basis.*^4^*”*

The last sentence indicates that it is the High Commissioner's opinion that the CRPD does not completely exclude involuntary treatment. If it were excluded altogether it would be seriously at variance with a widespread moral intuition (expressed in rights to life and health) that there are certain circumstances (including, for example, coma) in which treatment should be provided to a person who as a result of an impairment of mental functioning cannot make treatment decisions for himself or herself.

However, any criteria for involuntary treatment under the CRPD must be non-discriminatory and ‘disability-neutral’. We accept the position of the High Commissioner that when one arm of any set of necessary criteria for involuntary treatment is the presence of a ‘mental illness’ or ‘mental disorder’ (that is, a form of disability), unacceptable discrimination is introduced.

### 4.1 An example of a comprehensive DMC-based law

Dawson and Szmukler (2006) and Szmukler, Daw, and Dawson (2010) have proposed a form of law that is explicitly driven by the aim of removing discrimination against people with a mental illness. It is intended to apply to all persons, whatever their diagnosis (if they have one), whether they have a ‘mental disorder’ or not, who are unable through a lack of DMC to make a treatment decision for themselves. (We now use the term ‘decision-making capability’ (DMC) and not ‘capacity’ in order to make it clear that it is distinct from the term ‘legal capacity’). An impairment of DMC is not a ‘status’ attribute, that is, based on a diagnosis of a disorder or category of disability, but is a ‘functional’ attribute, that is, based on the inability to carry out a specific task at a specific time. Separate legislation authorising the civil commitment of ‘mentally disordered’ persons is argued to be unnecessary and discriminatory, and should be replaced by new, comprehensive legislation that would govern all non-consensual treatment. This new scheme described as the ‘Fusion Law’ proposal is based squarely on impaired decision-making capability principles: that is, on the impaired capability of a person to make a decision about treatment, from whatever cause — whether this be owing to schizophrenia, Alzheimer's disease, a confusional state — for example, post-operative, due to infection or after an epileptic seizure, a cerebrovascular accident, a head injury, or any other cause of a disturbance or impairment of mental functioning.

We will not in this paper venture in detail into the most appropriate criteria for the assessment of DMC. Szmukler, Daw and Dawson have opted for a modified version of the definition given in the English Mental Capacity Act 2005 (MCA). In relation to a treatment decision, DMC requires the ability to understand and retain information relevant to the decision, and the ability to ‘use, weigh or appreciate’ that information in the process of making that decision. This involves an appreciation that the information is relevant to the person's predicament, and the ability to use that information to generate and think through the consequences of having or not having the treatment in relation to the person's values and life choices. A criticism of this kind of test is that it is unduly ‘cognitive’, taking little account of emotion or values considerations when those are seen by most people, intuitively, as being important (see, for example, Tan, Hope, Stewart, & Fitzpatrick, 2006). The extent to which a test of DMC is necessarily ‘cognitive’ or ‘procedural’ is arguable. Banner (2010), for example, from a philosophical perspective, drawing on Donald Davidson's methods for ‘Radical Interpretation’, has argued that it is possible to take due account of a person's epistemic and evaluative commitments within such a framework while retaining a level of objectivity appropriate to the type of assessment required. This kind of approach involves an important dialogue between the assessor and the person whose DMC is being questioned. The assessor explores what appears to be an unusual belief or value affecting a treatment decision by expanding the discussion in an effort to reach an understanding of its coherence, or otherwise, within the wider system of ideas and values held by the person. A belief or value cannot be treated as an isolated attribute for assessment. Banner also goes on to discuss ways in which beliefs and values may come to be judged to lack coherence when viewed in this broader context, always accepting the fact that many of our ideas and decisions are far from perfectly constructed. Support for the person and consultation with others who know the person would enhance the assessment.

There may be a case for other criteria, for example, those presented by Bach and Kerzner (2010). They argue that the ability to express an intention (or will) and its coherence with a sense of a personal identity through time — that can be reliably discerned by an observer who knows the person well — can characterise a level of ‘decision-making capability’ that with support allows the person to make decisions that exercise legal capacity. This would be an example of ‘supported decision making’. Bach and Kerzner recognize another group of persons who lack decision-making capability within their broader definition, in whom their will and preferences cannot be adequately ascertained, and who they describe as requiring ‘facilitated’ decision-making, that is, by other persons, until the person's ‘will and preferences’, with the necessary supports, can be established.^5^

A precise definition is not necessary for the purposes of the argument presented in this paper. The important point is that at the time a person is required to make a specific treatment decision, they are not capable of doing so, according to a set of agreed criteria that are consistent with the principles of the CRPD. There is undoubtedly much conceptual and practical work still to be done on impaired DMC, but we see no other ethical basis for potentially intervening in a person's life when their wellbeing appears to be seriously threatened by what appears, at first sight at least, to be a seriously imprudent decision or an inability to make a decision at all.

The Fusion Law follows the MCA 2005 in requiring the person with impaired decision-making capability to participate as much as is possible in decision-making about the treatment. The person also is able to nominate a person to act as a decision maker if the patient should lose DMC. Such a person could, in practice, take on a supportive role. The Fusion Law also requires that an independent advocate be provided whose role is to support the person in having their voice heard. It would be entirely consistent with the principles of the fusion law proposal for it to be modified to incorporate a clear commitment to a ‘supported decision-making’ model.

Under the Fusion Law, involuntary treatment can be given to an objecting person only if there is a lack of DMC and, further, only if it is in the ‘best interests’ (as described below) of the person. The reason for the term ‘fusion’ in the law's description derives from the fact that while it is fundamentally based on impaired DMC (as in the MCA 2005), conventional capacity-based legislation fails to adequately regulate some important aspects of involuntary treatment — for example, how involuntary treatment, if it is in the ‘best interests’ of the person, is to be authorised, by whom, for how long, how is it to be reviewed, how often, what kind of appeals can be made, and so on. These aspects are spelt out in detail in conventional civil commitment legislation, and are in the Fusion Law, but are there merged with its DMC-based foundation.

While it is unnecessary to delve into notions of ‘best interests’ in great detail for the purposes of this paper, some important points need making. If decisions are needed when a person has seriously impaired DMC, some principles guiding those decisions are obviously necessary. There is some confusion about the meaning of terms in this area. We intend the following: the person who makes the decision is a ‘substitute decision maker’ (SDM) (or, perhaps better, following the proposals of Bach and Kerzner (2010), a ‘facilitator’ (F)); when a decision is made according to what the SDM or F believes the person would have made if they had DMC in the circumstances in which the decision has to be made, this would be a ‘substituted judgment’. This could also be expressed as a decision giving expression to the person's ‘will and preferences’. The term ‘best interests’ is used in the fusion proposal in a sense that develops from that used in the Mental Capacity Act (MCA) 2005: a complex judgement that is formed on the basis of a number of factors that the SDM must consider; these include the person's past and presently expressed wishes, beliefs and values (and in particular a relevant written statement made when the person had capacity), and factors that the person would have been likely to consider if they had been able to; and, consulting with relevant people in the person's life, including those nominated by the person, in order to assist in deciding what it is likely that the person would want. Under the MCA, the SDM must, so far as is reasonably practical, permit and encourage the person to participate, or to improve the person's ability to participate, as fully as possible in any act done or decision made. What we emphatically do not mean by ‘best interests’ is that the SDM or F decides on the basis of what he or she thinks is the right thing to do. The type of ‘best interests’ we propose is a ‘subjective’ best interests, that is, one that gives paramount importance to the values and preferences of the person.

While the MCA definition of ‘best interests’ has been generally well received, there remain a number of difficulties. The factors listed above having been considered, there is no guidance concerning how they are to be weighed (Dunn, Clare, Holland, & Gunn, 2007). The Court of Protection, to which cases falling under the MCA are referred, has attempted a balancing act between the person's expressed wishes and the seriousness of the harms that would occur, were those wishes acceded to. The court has so far maintained that although the person's past and current wishes and feelings are important factors to be taken into account when assessing best interests they cannot be determinative.^6^ The exception is an ‘advance decision’ to refuse treatment made when the person had decision-making capacity. The problem is most starkly played out where the person with a serious illness, especially when young, expresses a strong and persistent wish to be allowed to die (Richardson, 2013). However, a recent example of a ‘best interests’ approach recommended by the Victorian Law Reform Commission (2012) in Australia represents a development of the MCA principles and appears more in keeping with the CRPD. It is made clear that SDMs promote the “personal and social wellbeing” of the person when, as far as possible, they have paramount regard to making the judgments and decisions that the person would make themselves after due consideration if able to do so”. Consultation with others, the maximal participation of the person, and taking account of their cultural circumstances are also included. The full definition and guidance are presented in Appendix B.

There is certainly more work to be done on the basis for a decision when DMC is impaired, whether it be termed ‘best interests’, ‘subjective best interests’, or perhaps ‘best interests according to will and preference’, but the direction of travel is becoming clear.

It is interesting that in the ‘will and preferences’ approach of Bach and Kerzner, DMC and ‘best interests’ are, in effect, merged. Assessment of DMC is entailed in the attempt to establish what are the person's ‘will and preferences’ in relation to a decision, while ‘best interests’, as we would conceive it, is furthered by facilitating actions that accord with the person's ascertained ‘will and preferences’. Furthermore, within their schema, one could argue that the facilitated decision is not a ‘substituted’ one in an important sense, since it gives expression to the person's ascertained ‘will and preferences’.

A further point is worth noting in relation to patients who experience episodes of psychosis, but who recover in between, indeed the most common course taken by such illnesses. Here we have the inestimable advantage — in comparison with end-of-life conditions — of being able to discuss with patients, in the light of already experienced episodes of illness, what their preferences would be if a relapse were to occur. These might change over time; the person could ‘fine-tune’ their preferences if they were to experience a further episode. There is accumulating evidence that this kind of advance statement or joint crisis planning can reduce the use of coercive interventions (Henderson et al., 2004, Swanson et al., 2008).

## 5 Impaired capability and disability

A loss of DMC, (which should specifically include the inability of supported decision-making to help the person make a decision) is the necessary entry criterion to consideration for involuntary treatment under the Fusion Law proposal. As it is argued this law does not entail discrimination against those with a disability, it is thus consistent with the requirements of the CRPD. Important in this regard is the fact that the Fusion Law applies to all persons, no matter what the cause of the impairment of DMC, whether it is a so-called mental illness or physical illness. Furthermore, it applies equally to a person who does not have a pre-existing condition associated with a disability as to one who does — for example, equally to the person with a diagnosis of schizophrenia who loses DMC during a relapse of psychosis as to the previously fit young person who suffers a head injury in a motor car accident.

An objection might be raised that loss of DMC due to a disturbance or impairment of mental functioning itself comprises a ‘disability’, and thus could not form a valid, ‘disability-neutral’, criterion under the CRPD for overriding a person's wishes. Thus, according to this argument, the ‘disability’, that is the loss of DMC, itself, would become the reason for involuntary treatment and is on that account discriminatory and thus incompatible with the CRPD (Bartlett, 2012). The problem may be most troublesome in cases where a severe impairment of capability to make treatment decisions may be more or less permanent, as in persons with very severe intellectual disability or dementia.

Let us examine this objection. An impairment of DMC is specific to a particular decision that is necessary at a particular time. The notion of ‘blanket’ incapacity is, as the discussion of DMC above should make clear, entirely rejected. In the vast majority of people with a mental illness, even where the illness is severe and long-term, DMC is intact for most, or indeed all, of the time. It is usually at times of relapse only that it may become impaired in people with, for example, schizophrenia or bipolar disorder.

Even then, patients with a mental illness severe enough to result in admission to acute wards of a psychiatric hospital, over 40% retain DMC (Owen et al., 2008, Owen et al., 2009).

In persons with mild to moderate dementia, DMC may be present most of the time, but be compromised on occasions by inter-current illness such as a urinary tract infection or an adverse reaction to medication for an unrelated disorder. As stated earlier, loss of DMC may occur in any person, whether they have a (pre-existing) condition associated with a disability or not. It may be present for minutes (as in a hypoglycaemic reaction in a person with diabetes), hours, days, or much longer. Also, as mentioned earlier, DMC is ‘functionally’ assessed, and is not based on ‘status’ (such as having a diagnosis of ‘schizophrenia’).

Thus, taking some concrete examples, we suggest that each of the following instances in which a person, despite support (or offers of support that are refused), continues to adamantly reject treatment as a result of an impairment of DMC can be regarded as, in principle, the same; and, in each case non-consensual treatment could be justified if it were in accord with a form of subjective best interests, for example, following the principles outlined in Appendix B:

**Table:** 

1	A person with no physical or mental illness	is struck by a car and sustains a severe head injury causing a haemorrhage that will result in death without surgical intervention.
2	A person with a psychosis, stable and experiencing no symptoms	
3	A person with a psychosis, with obvious severe symptoms	
4	A person with an intellectual disability	
5	A person with previously good mental abilities develops a delirium as a result of the adverse effects of drug treatment for heart failure	and refuses to eat or drink because he is convinced all food given to him is poisoned with the intention to kill him.
6	A person with a psychosis, usually stable, develops a delirium as a result of the adverse effects of drug treatment for heart failure	
7	A person with a psychosis, usually stable and with good mental abilities, suffers a relapse of the psychosis due to ‘crack’ cocaine use	
8	A person with a psychosis, usually stable and with good mental abilities, suffers a relapse of the psychosis of unknown cause	
9	A person with an intellectual disability who normally enjoys his food, after being informed that he has a lymphoma, a life threatening disease, becomes psychotic	

These cases, we argue, illustrate why there is good reason to separate impaired DMC from ‘disability’ even though both may be present at the same time. They show that each may occur independently, one in the absence of the other. They are conceptually distinct. In this sense, then, DMC can claim to be ‘disability neutral’ and a valid criterion under the CRPD (in association with an appropriate test of subjective best interests or ‘will and preferences’) for involuntary treatment. Such treatment would be provided to those with disabilities ‘on an equal basis with others’. Indeed, failure to provide treatment for a serious condition that is rejected as a result of a person's impaired DMC would be in conflict with Article 25 of the CRPD:

*‘States Parties recognize that persons with disabilities have the right to the enjoyment of the highest attainable standard of health without discrimination on the basis of disability’.*

If non-consensual treatment is to be allowed, for example, in case 1 above, but not for 2, 3 or 4, then such discrimination is clearly a consequence; or in case 5, but not 6. If allowed for cases 5 and 6, why not for 7 and 8?

It seems unlikely that an intention behind the CRPD was to exclude the possibility of all forms of non-consensual treatment in all cases, including semi-coma or coma (which indeed would ensue in each of the cases above if treatment were to be withheld). One draft^7^ of Article 17, which was dropped after strong resistance from disability organisations (Bartlett, 2012), included the following:

2. States Parties shall protect persons with disabilities from forced intervention or forced institutionalisation aimed at correcting, improving or alleviating any actual or perceived impairment.
3. In cases of medical emergency or issues of risk to public health involving involuntary interventions, persons with disabilities shall be treated on an equal basis with others.
4. States Parties shall ensure that involuntary treatment of persons with disabilities is:
  (a) Minimized through the active promotion of alternatives;
  (b) Undertaken only in exceptional circumstances, in accordance with procedures established by law and with the application of appropriate legal safeguards;
  (c) Undertaken in the least restrictive setting possible, and that the best interests of the person concerned are fully taken into account;
  (d) Appropriate for the person and provided without financial cost to the individual receiving the treatment or to his or her family.

One can understand the difficulties, especially with (4), which appears to be discriminatory, but the explicit reference to medical emergencies seems to have been lost in the final text of the CRPD. Article 17 has been left as a bald single sentence.

Article 25 (d) requires:

*‘health professionals to provide care of the same quality to persons with disabilities as to others, including on the basis of free and informed consent by, inter alia, raising awareness of the human rights, dignity, autonomy and needs of persons with disabilities through training and the promulgation of ethical standards for public and private health care’;*

It would seem unreasonable to interpret 25 (d) as completely excluding involuntary treatment — under any circumstances, for anyone — including case 1 above. If it is to be permitted for case 1, would it not be a failure to provide ‘care of the same quality’ for cases 2, 3, 4, and so on?

## 6 Conclusions

The CRPD poses major challenges for a justification for involuntary treatment that is not discriminatory. We welcome these challenges as we maintain that existing mental health legislation is unfairly discriminatory against people with a mental illness. Some have stated that the CRPD may effectively rule out involuntary treatment. However, we suggest that very few would support the idea that the state never, even as a last resort, has a duty to protect those who are clearly unable to make crucial treatment decisions for themselves. We suggest that an impaired decision-making capability approach, as in the Fusion Law proposal, offers a non-discriminatory basis for involuntary treatment where attempts at supported decision-making have proven unsuccessful. If it is accepted that impaired DMC can be ‘disability-neutral’, as we have argued, and that it may provide the gateway to a consideration of involuntary treatment, it cannot on its own justify involuntary treatment. There still remains the thorny question of what further justification is required. A version of subjective best interests where the patient's perspective is paramount is the most appropriate basis for a decision and aims to give expression to the person's ‘will and preferences’. If the person's will and preferences are endorsed and acted upon in this manner, in an important sense, the decisions made are not ‘substituted’ ones. Arriving at what constitutes the person's ‘real’ or ‘authentic’ will and preferences when there is a difference between those currently expressed as opposed to those previously expressed may present difficulties. There is clearly still much conceptual and practical work to be done in developing these ideas.

## Appendix A Articles of the Convention on the Rights of Persons with Disabilities relevant to the discussion of involuntary treatment

### Article 12

#### Equal recognition before the law

1. States Parties reaffirm that persons with disabilities have the right to recognition everywhere as persons before the law.
2. States Parties shall recognize that persons with disabilities enjoy legal capacity on an equal basis with others in all aspects of life.
3. States Parties shall take appropriate measures to provide access by persons with disabilities to the support they may require in exercising their legal capacity.
4. States Parties shall ensure that all measures that relate to the exercise of legal capacity provide for appropriate and effective safeguards to prevent abuse in accordance with international human rights law. Such safeguards shall ensure that measures relating to the exercise of legal capacity respect the rights, will and preferences of the person, are free of conflict of interest and undue influence, are proportional and tailored to the person's circumstances, apply for the shortest time possible and are subject to regular review by a competent, independent and impartial authority or judicial body. The safeguards shall be proportional to the degree to which such measures affect the person's rights and interests.
5. Subject to the provisions of this article, States Parties shall take all appropriate and effective measures to ensure the equal right of persons with disabilities to own or inherit property, to control their own financial affairs and to have equal access to bank loans, mortgages and other forms of financial credit, and shall ensure that persons with disabilities are not arbitrarily deprived of their property.

### Article 14

#### Liberty and security of the person

1. States Parties shall ensure that persons with disabilities, on an equal basis with others:
  (a) Enjoy the right to liberty and security of person;
  (b) Are not deprived of their liberty unlawfully or arbitrarily, and that any deprivation of liberty is in conformity with the law, and that the existence of a disability shall in no case justify a deprivation of liberty.
2. States Parties shall ensure that if persons with disabilities are deprived of their liberty through any process, they are, on an equal basis with others, entitled to guarantees in accordance with international human rights law and shall be treated in compliance with the objectives and principles of this Convention, including by provision of reasonable accommodation.

### Article 17

#### Protecting the integrity of the person

Every person with disabilities has a right to respect for his or her physical and mental integrity on an equal basis with others.

### Article 25

#### Health

States Parties recognize that persons with disabilities have the right to the enjoyment of the highest attainable standard of health without discrimination on the basis of disability. States Parties shall take all appropriate measures to ensure access for persons with disabilities to health services that are gender-sensitive, including health-related rehabilitation. In particular, States Parties shall:

(a) Provide persons with disabilities with the same range, quality and standard of free or affordable health care and programmes as provided to other persons, including in the area of sexual and reproductive health and population-based public health programmes;
(b) Provide those health services needed by persons with disabilities specifically because of their disabilities, including early identification and intervention as appropriate, and services designed to minimize and prevent further disabilities, including among children and older persons;
(c) Provide these health services as close as possible to people's own communities, including in rural areas;
(d) Require health professionals to provide care of the same quality to persons with disabilities as to others, including on the basis of free and informed consent by, inter alia, raising awareness of the human rights, dignity, autonomy and needs of persons with disabilities through training and the promulgation of ethical standards for public and private health care;
(e) Prohibit discrimination against persons with disabilities in the provision of health insurance, and life insurance where such insurance is permitted by national law, which shall be provided in a fair and reasonable manner;
(f) Prevent discriminatory denial of health care or health services or food and fluids on the basis of disability.

## Appendix B

‘Recommended decision-making principles’: from Victorian Law Reform Commission *Guardianship: Final Report* (2012) p.399

Legislation should require substitute decision makers to exercise their powers in a manner that promotes the personal and social wellbeing of the represented person.

Substitute decision makers promote the personal and social wellbeing of the person when, as far as possible, they:

(a) have paramount regard to making the judgments and decisions that the person would make themselves after due consideration if able to do so
(b) act in consultation with the person, giving effect to their wishes
(c) support the person to make or participate in decisions
(d) act as an advocate for the person, and promote and protect their rights and dignity
(e) encourage the person to be independent and self-reliant
(f) encourage the person to participate in the life of the community
(g) respect the person's supportive relationships, friendships and connections with others
(h) recognise and take into account the person's cultural and linguistic circumstances
(i) protect the person from abuse, neglect and exploitation.

Additional guidance for substitute decision makers:

In determining the judgments and decisions a represented person would make after due consideration, substitute decision makers should be guided by:

(a) the wishes and preferences the person expresses at the time a decision needs to be made, in whatever form the person expresses them
(b) any wishes the person has previously expressed, in whatever form the person has expressed them
(c) any considerations the person was unaware of when expressing their wishes which are likely to have significantly affected those wishes
(d) any circumstances that have changed since the person expressed their wishes which would be likely to significantly affect those wishes
(e) the history of the person, including their views, beliefs, values and goals in life.

## Footnotes

1 Annual report of the High Commissioner for HumanRights to the General Assembly. A/HRC/10/49, presented 26 January 2009, para 48-9. Available at http://www.ohchr.org/EN/UDHR/Documents/60UDHR/detention_infonote_4.pdf. Accessed 26 July 09

2 Committee on the Rights of Persons with Disabilities. Sixth Session 19-23 Sept 2011. Concluding observations.

3 Committee on the Rights of Persons with Disabilities. Fifth Session 11-15 April 2011. Concluding observations.

4 Annual report of the High Commissioner for HumanRights to the General Assembly. A/HRC/10/49, presented 26 January 2009, para 48-9. See also UN, Office of the High Commissioner for Human Rights ‘Persons with Disabilities’ Dignity and Justice for Detainees Week, Information Note No. 4, (2008) p. 2. Available at http://www.ohchr.org/EN/UDHR/Documents/60UDHR/detention_infonote_4.pdf. Accessed 26 July 09.

5 The criteria for ‘supported’ and ‘facilitated’ decision-making presented by Bach and Kerzner appear to be highly appropriate, respectively, for persons with an intellectual disability and for persons in a coma or an extremely impaired cognitive state. The extent to which they are applicable to people with mental illness, who may have an apparently fixed delusional idea or who feel compelled to respond to hallucinations, for example, is not clear, but certainly merits investigation.

6 For an example, see Munby J, *In Re M (Statutory Will)* [2009] EWHC 2525 (Fam)

7 Ad Hoc Committee, Report of the Seventh Session, A/AC.265/2006/2 (13 Feb 2006).
